# Desire for Information and Preference for Participation in Treatment Decisions in Patients With Cancer Presenting to the Department of General Surgery in a Tertiary Care Hospital in India

**DOI:** 10.1200/JGO.17.00144

**Published:** 2018-02-13

**Authors:** Sushmitha Dharani Sankar, Baskaran Dhanapal, Gomathi Shankar, Balamourougan Krishnaraj, Sandhya Karra, Vignesh Natesan

## Abstract

**Purpose:**

Providing appropriate information to patients about their illness helps them to cope with the diagnosis. Shared decision making is a key concept in managing patients with cancer. There are no data available about the desire for information and preference for participation in treatment decisions among Indian patients with cancer. The objective of this study was to estimate the proportion of patients who have information needs and to study the patient preference for participation in treatment decisions and the factors associated with them.

**Methods:**

A cross-sectional survey was conducted among patients with cancer older than 18 years. They were interviewed with a questionnaire after signing an informed consent. The association of sex, educational level, residence, diagnosis (type of cancer), Eastern Cooperative Oncology Group performance status, and treatment status with information needs and decision-making preference was analyzed using χ^2^ test

**Results:**

Approximately 81% of patients said that they had an absolute need to know if the illness was cancer, and > 70% of patients either had an absolute need to know or would like to know about the prognosis, treatment options, and adverse effects. Regarding the decision-making preferences, 97% wanted their treating physicians to make the decision regarding their treatment, and 66% preferred to share decision making with their family.

**Conclusion:**

The majority of the patients with cancer expressed a need for knowing whether they had cancer. When it comes to treatment decisions, most of them preferred a passive role, and the majority wanted to involve their families in the decision-making process. We recommend that the treating physician should elicit the patient’s preference in participating in treatment decisions and their preference about involving their family in making treatment decisions

## INTRODUCTION

Diagnosis of cancer can be a stressful experience for patients. Providing appropriate information to patients about their illness helps them to cope with the diagnosis.^1^ A patient who is diagnosed with cancer has to decide about multiple treatment options for the same illness. Some of the treatment options have an established benefit, but the benefit may be uncertain in some. Hence, making an appropriate treatment decision is essential to improve outcome

Shared decision making is seen as a key concept in managing patients with cancer. The process of decision making is complex and is an outcome of the interaction between the patient, their family, and the treating physician. The relative roles of each of these three key participants vary in different cultures.^2^ It has been shown that in the West, patients prefer an active role in making treatment decisions, and patients who had active role had better outcomes. But, in India, physicians often play a paternalistic role in decision making, and family members also play a major role in health-related decisions. We cannot assume that patients will be passive, active, or collaborative, because patients are a heterogeneous group, and everyone is likely to have their own preferences. To be sensitive to the needs and expectations of the patients, one needs to establish the actual patient preference in decision making

Patients need appropriate information to cope with the diagnosis of cancer and to make informed choices about their treatment. However, the concept of appropriate information differs among different cultures. In a study done in Leicester, the need for additional information was different among Asian and white patients.^3^

We designed our study with the following objectives. First, we sought to estimate the proportion of patients with cancer who have information needs and to categorize the various needs as per the information needs questionnaire. The second objective was to study the patient’s preference for participation in treatment decisions. Third, we examined the patient’s preference regarding the role of their family members in treatment decisions and the factors influencing the information needs of the patients and their preference for participation in treatment decisions.

## METHODS

The study was conducted in the Department of General Surgery, Jawaharlal Institute of Postgraduate Medical Education and Research (JIPMER), Pondicherry, India. JIPMER is a 2,000-bed tertiary care referral center in Pondicherry, India. An average of 4,000 patients with malignancies involving different systems present to the surgery cancer clinic every year This was a cross-sectional study done from February 2017 to August 2017, after approval from the Institute Ethics Committee (Human Studies), JIPMER. The inclusion criterion was all patients with cancer older than 18 years attending the department of general surgery, JIPMER. The exclusion criteria were patients with cancer presenting to emergency with bleeding, obstruction, or perforation and terminally ill patients with cancer.

The sample size was calculated using OpenEpi, Version 3.01. Assuming that 87% of patients with cancer would have a preference to know all possible information about their disease and with the absolute precision as 6%, and CI as 95%, the number of patients needed was 121. Allowing a 10% dropout rate, the final sample size was 133 patients.

### Data Collection

The data were collected by administering a questionnaire by an interviewer after obtaining an informed consent from the eligible patients. The interviewer was a medical student in JIPMER. The interviews were conducted in the cancer clinics and in the general surgery wards, at a time that was convenient for the interviewer and the patient.

First, the patients were asked about their overall preference for information in general. Then, subsequent specific questions were asked to elicit the patients’ attitudes to receive information about particular aspects of their illnesses and treatment. The patients were asked to indicate if they “absolutely need to know” or “would like to know” or “prefer not to know,” regarding each of the specific aspects.

Then, the patients were questioned regarding their preference to participate in making treatment decisions and were asked to select one of the following responses:

- I prefer to make the final selection about which treatment I will receive
- I prefer to make the final selection of my treatment after seriously considering my doctor’s opinion
- I prefer that my doctor and I share responsibility for deciding which treatment is best for me
- I prefer that my doctor makes the final decision about which treatment, but seriously considers my opinion
- I prefer to leave all decisions regarding treatment to my doctor

Finally, the patients were questioned regarding their preference to involve family members in making treatment decisions and were asked to select one of the following responses:

- I prefer to make the final selection about which treatment I will receive
- I prefer to make the final selection of my treatment after seriously considering my family’s opinion
- I prefer that my family and I share responsibility for deciding which treatment is best for me
- I prefer that my family makes the final decision about which treatment, but seriously considers my opinion
- I prefer to leave all decisions regarding treatment to my family

Statements A and B were considered reflective of an active role, the third statement (C) was considered to indicate preference for a collaborative role, and the last two statements (D and E) reflected a passive role. Data regarding the patient’s educational level, occupation, marital status, residence, monthly family income, diagnosis (type of cancer), Eastern Cooperative Oncology Group (ECOG) performance status, and treatment status were obtained from their case records.

### Statistical Analysis

Categorical variables like sex, educational level, occupation, marital status, residence, diagnosis (type of cancer), ECOG performance status, comorbid illnesses, treatment status, information needs, and decision preference were expressed as proportions. The association between sex, educational level, residence, diagnosis (type of cancer), ECOG performance status, and treatment status and information needs and decision-making preference was analyzed using χ^2^ test. A *P* value < .05 was considered statistically significant. The statistical analysis was done using IBM SPSS version 20.0 statistical software (SPSS, Chicago, IL).

## RESULTS

A total of 133 patients out of 137 who were approached agreed to participate in our study. The mean age of the study participants was 49.7 years. Thirty-one participants (23.30%) were ≤ 40 years of age, and 102 participants (76.69%) were older than 40 years. Ninety-four participants (70.67%) were women, and 39 participants (29.32%) were men. The majority of the patients (73.68%) were from a rural area. Of all the participants, only three were graduates. Although 36 patients (27.06%) did not have any formal education, 82 (61.65%) had attended primary school, and 12 (9.02%) had attended high school. Forty-nine patients (36.8%) were unemployed. Breast cancer (47.36%) was the predominant diagnosis among the study participants, followed by gastric cancer (18.79%) and rectal cancers (12.03%). Although the performance status of 79 patients (59.39%) was ECOG 2, 25 patients (18.79%) were in ECOG 1, and 29 patients (21.80%) were in ECOG 3. Among the participants, 127 (95.48%) were diagnosed with their illness in the past 1 year. Fifty patients (37.59%) were awaiting treatment, 76 patients (57.14%) were undergoing treatment, and seven patients (5.26%) had completed treatment and were on follow-up visits (Table 1).

**Table 1:**
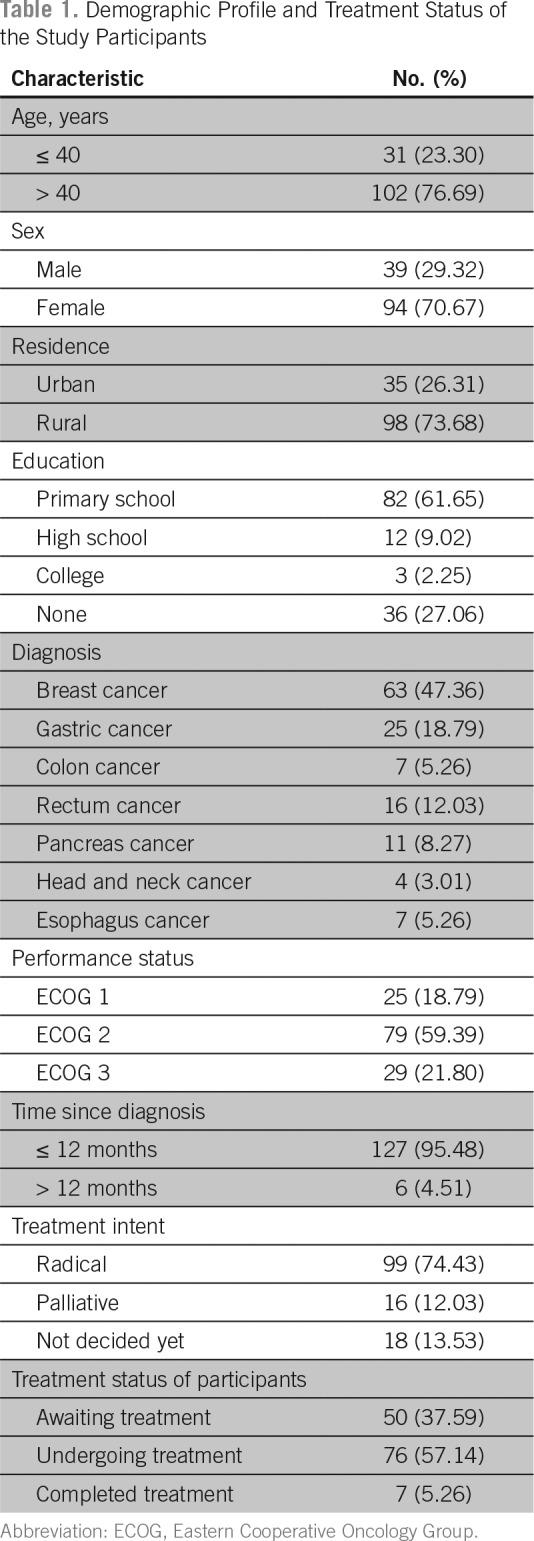
Demographic Profile and Treatment Status of the Study Participants

Among the participants, 107 (80.4%) expressed that they have the need for information regarding their illness. Although 68% preferred to have as much information as possible, 27% preferred to know only if it was something good, and 5% preferred not to know anything about their illness (Table 2). When asked about their desire for information regarding specific aspects of their illness, 108 patients (81.20%) said that they had an absolute need to know if the illness was cancer. The proportion of patients who had a desire to absolutely know about the medical name of the illness, progression of the disease, chances of cure, treatment options available, the adverse effects of treatment, and how the treatment works were 18.7%, 38.3%, 40.6%, 40.6%, 35.3%, and 21.8%, respectively. The proportion of patients who expressed that they prefer not know about the specific aspects of their illness was < 30% in each of the above categories (Table 3).

**Table 2:**
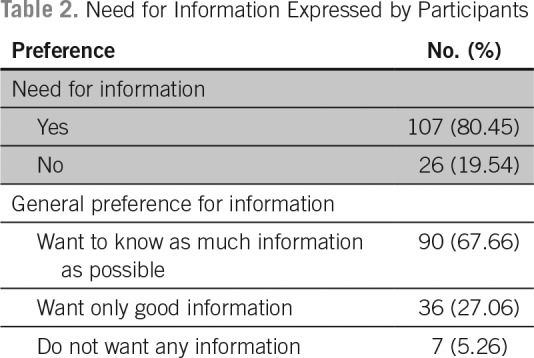
Need for Information Expressed by Participants

**Table 3:**
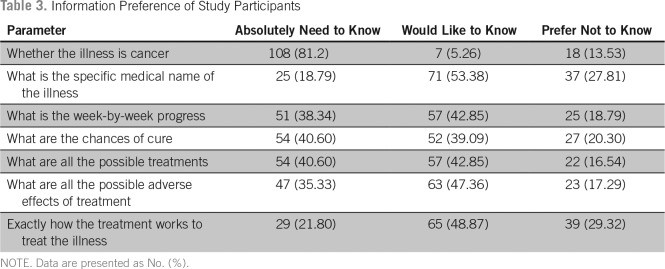
Information Preference of Study Participants

Regarding the preference to participate in making treatment decisions, 129 patients (96.9%) preferred a passive role, two patients (1.5%) preferred an active role, and two (1.5%) preferred a collaborative role (Table 4). Although 88 participants (66.16%) preferred to share the responsibility of making treatment decision with their family, 17 (12.78%) preferred to leave the responsibility to their family members, and 28 (21.05%) preferred to make the decision on their own (Table 5).

**Table 4:**
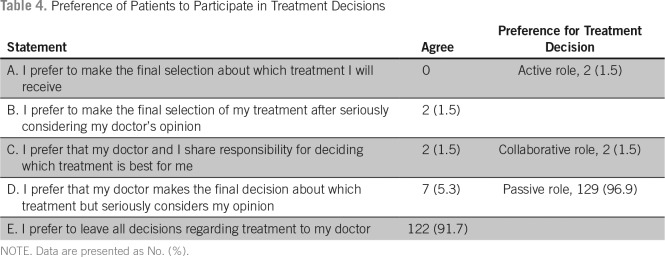
Preference of Patients to Participate in Treatment Decisions

**Table 5:**
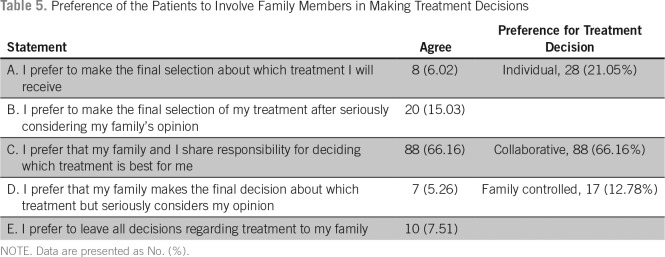
Preference of the Patients to Involve Family Members in Making Treatment Decisions

There was no significant association between the demographic variables, diagnosis, time since diagnosis, or treatment status and the preference of the patient to participate in treatment decisions (Table 6). The family involvement scale also did not show any significant association with demographic variables, diagnosis, performance status, and time since diagnosis. Patients who were awaiting treatment preferred a collaborative decision with their family compared with those who were undergoing treatment or those who have completed treatment (Tables 7 and 8).

**Table 6:**
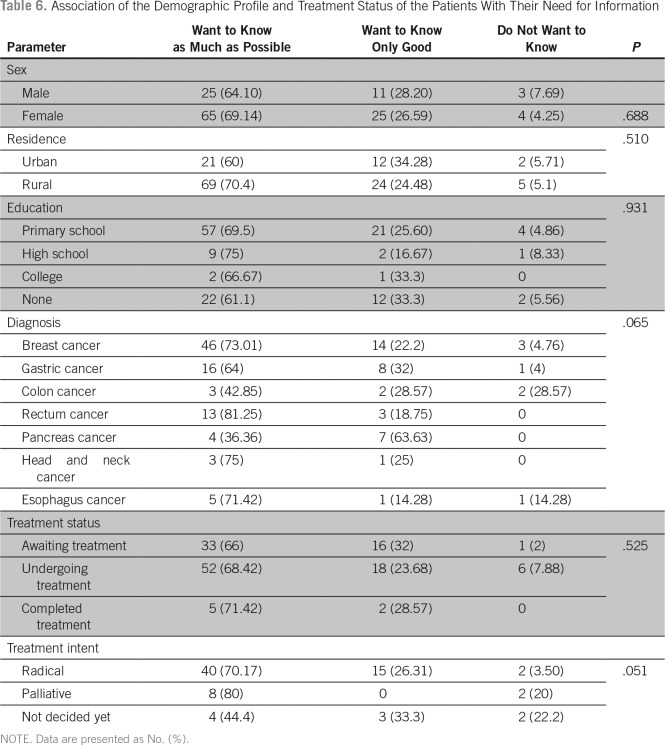
Association of the Demographic Profile and Treatment Status of the Patients With Their Need for Information

**Table 7:**
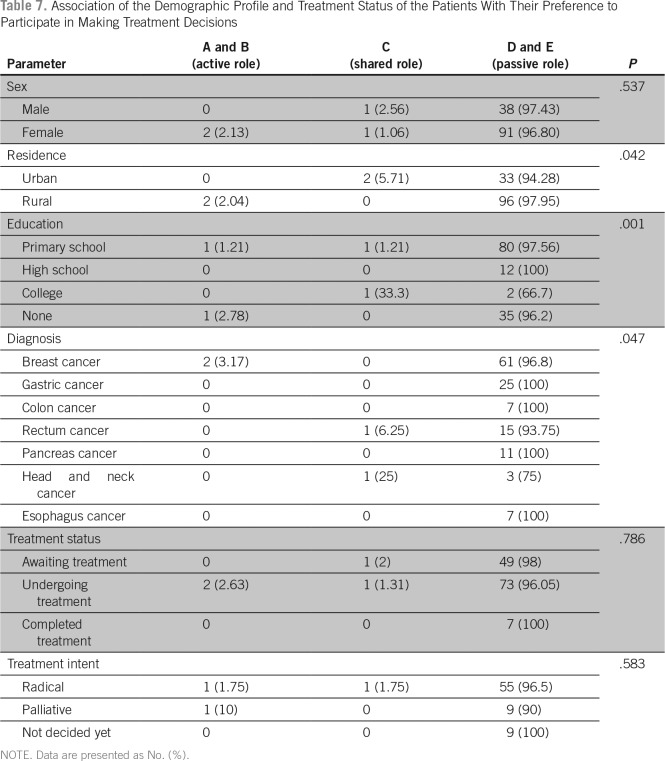
Association of the Demographic Profile and Treatment Status of the Patients With Their Preference to Participate in Making Treatment Decisions

**Table 8:**
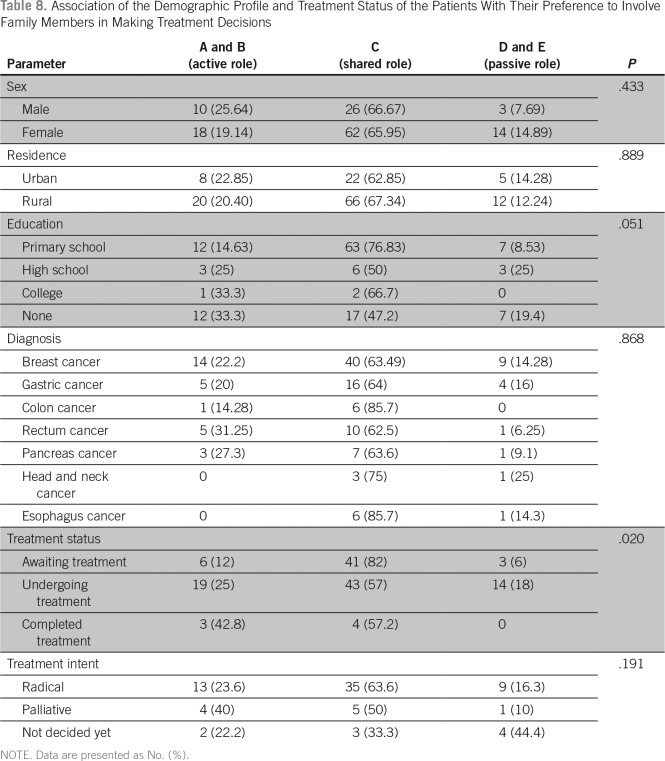
Association of the Demographic Profile and Treatment Status of the Patients With Their Preference to Involve Family Members in Making Treatment Decisions

## DISCUSSION

There is an increase in awareness among health care professionals throughout the world to encourage their patients to participate in making treatment decisions. This process begins with providing appropriate information to patients. There is a dearth of knowledge regarding what Indian patients want to know regarding their illness and what are their decision-making preferences. Family participation in decision making and communication is an important factor in India.^4^ Nearly 77% of Indian families did not want to disclose the information about cancer to the patient.^5^ The information needs can also vary with age, diagnosis, educational status, and intent of treatment.^6^

In our study, the majority of the patients expressed a desire for information regarding their illness and treatment. Factors such as the patient’s age, sex, education status, residence, treatment intent, and treatment received did not affect the information needs of the patients. Although there are many studies done in the West to assess the information needs of the patients with cancer, there has only been one such study done in India.^5^ The results of that study indicate a significant association between the age of the patient, level of education, and type of treatment with their information needs. The authors concluded that most of the patients wanted to know about their illness and treatment, and the desire for information is significantly greater among younger patients, literate patients, and those receiving treatment with a curative intent.^5^ Although the majority of our patients had a lower level of education, we found that most of them desired to know about their illness and treatment.

Approximately 97% of our study participants preferred a passive role in making their treatment decisions, with nearly 91% preferring their treating doctor to make all decisions. Only 1.5% of participants preferred an active role. This is in stark contrast to the studies done in the United States and in other developed countries, where a significant number of patients preferred an active role. In a meta-analysis by Singh et al,^7^ the authors found that 50% of patients preferred a collaborative role with their physicians. A study by Bruera et al^8^ showed that 63% of patients preferred shared decision making, 20% preferred an active role, and only 17% preferred to be passive. Similar results were obtained in the study done by Schaede et al^9^ in Japan.

The preference for a passive role by our patients could be due to various reasons. It has been shown that Indian patients place a higher value on their health than the principle of their personal autonomy.^10^ This also could be because the majority of the study patients had a lower level of education. There are studies that showed that patients with a lower level of education preferred a passive role in treatment decisions.^9,11^ One of the reasons for the patients with a low level of education to prefer a passive role may be their lack of awareness about the disease and the treatment options. It has been shown that patients prefer an active role when the trust levels with physicians are less.^12^ Traditionally, Indian patients are used to a paternalistic approach by their physicians and have a higher degree of trust in their physicians. This could be one more reason that we had a large number of patients who preferred leaving treatment decisions to their physicians.

It has been shown that aligning the patients’ expectations with their actual experiences results in greater degree of patient satisfaction. In our study, we had looked only at the preferred role and not the actual role in decision making. To avoid decision conflicts, it seems prudent to elicit the preferred role of Indian patients rather than assuming that they will prefer a shared role like their counterparts in other nations.

The diagnosis did not have any significant impact on the decision-making preference. Two patients out of 63 patients with breast cancer preferred an active role. One patient with rectal cancer and one patient with head and neck malignancy preferred shared decision making with their physician. Patients with other diagnoses preferred a passive role. This may be because of the body image changes associated with the treatment of these malignancies (mastectomy, ostomy, or flaps), whereas patients may not feel any body image changes as a result of treatment of gastric cancer, pancreatic cancer, or colonic cancer. There was no significant association between demographic variables such as age, sex, residence, or treatment status and decision-making preference.

Approximately 66% of our patients preferred to share the responsibility of making a treatment decision with their family, 17 (12.78%) preferred to leave the responsibility to their family members, and 28 (21.05%) preferred to make the decision on their own. Family involvement has been shown to be associated with improved outcomes in patients with cancer. Involving family members in the treatment planning has been shown to increase patient satisfaction. There are no data available about family involvement in treatment decisions among Indian patients with cancer. A large population-based cohort study involving recently diagnosed lung or colorectal cancer in North America showed that only 1.5% had family-controlled decisions about their cancer treatment.^13^ This is in contrast to our results, where 12.78% had family-controlled decisions. The North American study identified that family-controlled decisions were more common among non–English-speaking Asians. In the Indian tradition and culture, the institution of family plays a major role over the individual. It is seen that often a responsible family member makes most health-related decisions for the other family members. This may be the reason a majority our patients preferred a shared role with their family, and a significant number wanted their families to be the sole decision-making authority. The decision to involve family in decisions did not have a significant association with demographic variables, diagnosis, performance status, and time since diagnosis. Patients who were awaiting treatment preferred a collaborative decision with their family when compared with those who were undergoing treatment or those who have completed treatment.

The strength of our study is that this the first study, to our knowledge, to examine the decision-making preferences of Indian patients with cancer. There are a few limitations to our study. First, the survey was conducted in a single institution in South India. Most of our study participants were from a rural area and had poor levels of education. Hence, there is a poor representation of people with a higher socioeconomic status. The study results may not be generalizable to the whole of India. However, the majority of Indians live in rural areas and have a poor socioeconomic background. Thus, we presume that the decision preferences may be similar for most Indian patients. Second, we had used a cross-sectional study design, wherein we had interviewed patients at a single point in time. There are studies that have found that patient preferences are dynamic and that patients have the tendency to change their preferences.^14^ Third, 96.9% of patients preferred a passive role and let their physicians decide for them. There may be an element of social desirability bias that could have influenced the answers, because the patients knew that the interviewer was a trainee physician.

Despite the limitations, our study offers three interesting insights about the decision-making choices and information needs of Indian patients with cancer. First, the majority of patients want to know more about their illness, contrary to the usual assumptions. Second, even if they want to know more about their illness, they prefer that their physicians should decide their treatment, which is in stark contrast to Western studies. Third, the majority prefer to involve their family in treatment decisions when compared with other populations.

In conclusion, Indian patients with cancer prefer a passive role in treatment decision making and prefer to share the responsibility with their family. But still, they had a strong desire for information regarding their illness and treatment. We recommend that the treating physician should elicit the patient’s preference in participating in treatment decisions and their preference about involving their family in making treatment decisions. We suggest that the physicians should disclose the information regarding the illness to the patients and discuss the treatment options with them, along with their family members. Additional multi-institutional prospective studies are required to assess the information needs and decision-making preferences among patients representing all socioeconomic strata.
